# La dermatomyosite paranéoplasique révélant un carcinome indifférencié du nasopharynx: à propos d'un cas

**DOI:** 10.11604/pamj.2016.24.29.4822

**Published:** 2016-05-09

**Authors:** Fatima Zahra Ziani, Sami Aziz Brahmi, Rajae Najib, Rajae Kanab, Samia Arifi, Fatima Zahra Mernissi, Nawfal Mellas

**Affiliations:** 1 Service d'Oncologie Médicale du Centre Hospitalier Universitaire Hassan II, Fès, Maroc; 2 Service de Dermatologie du Centre Hospitalier Universitaire Hassan II, Fès, Maroc

**Keywords:** Dermatomyosite, cancer, cancer du cavum, Dermatomyositis, cancer, nasopharyngeal cancer

## Abstract

La dermatomyosite (DM) est une maladie inflammatoire d'origine inconnue qui se manifeste sous forme de myopathie associée à lésions cutanées typiques. L'association DM et cancer est fréquente (18 a 32% selon les séries). Décrite pour la première fois par Stertz en 1916 en association avec un cancer gastrique. Tous les types histologiques et toutes les localisations de cancers observés danss la population générale peuvent être associés à la DM. Son association avec le carcinome nasopharyngé (NPC) est peu décrite et de l'ordre d'un cas pour 1000 cas de cancer nasopharyngé. Nous rapportons une observation de dermatomyosite révélant un cancer du nasopharynx localement avance.

## Abstract (en)

Dermatomyositis (DM) is an inflammatory disease of unknown origin that manifests as a myopathy associated with typical skin lesions. Association between DM and cancer is frequent (from 18% to 32% according to case series). It was described for the first time by Stertz in 1916 in association with gastric cancer. All histological types and sites of cancer in the general population may be associated with DM. Its association with nasopharyngeal carcinoma (NPC) is rarely described and the incidence proportion is 1 case of nasopharyngeal carcinoma per 1.000 persons.

## Introduction

La dermatomyosite (DM) est une connectivité rare qui associe une atteinte cutanée caractéristique et une atteinte musculaire prédominant aux ceintures. Elle s'associe dans 18 à 32% des cas à une néoplasie sous jacente apparaissant avant, simultanément, ou après le diagnostic du cancer [1]. L'incidence de DM associée avec les carcinomes nasopharyngés est de l'ordre d'un pour 1000 cas de cancer nasopharyngé [2]. Nous rapportons une observation sur une dermatomyosite paranéoplasique révélant un carcinome indifférencie de nasopharynx.

## Patient et observation

**Figure 1:**
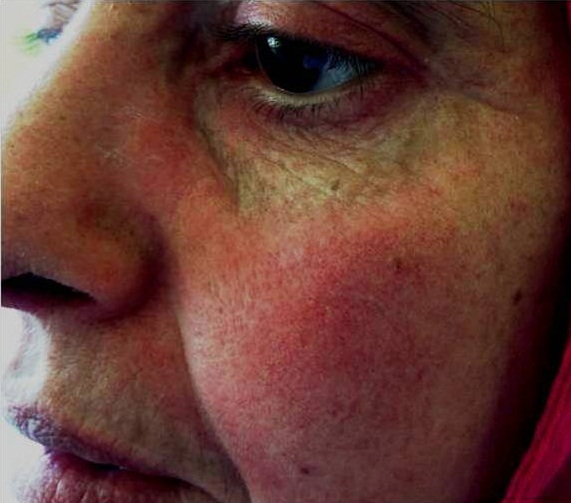
Éruption cutanée erythémateuse au niveau du visage

**Figure 2:**
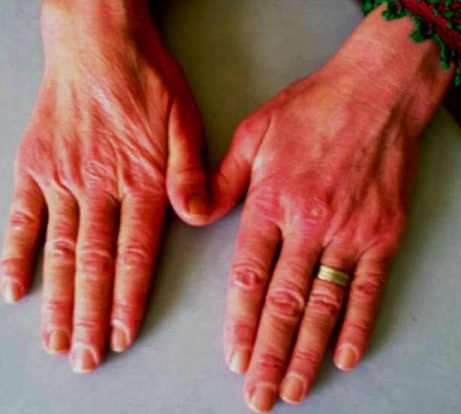
Éruption cutanée erythémateuse au niveau des mains

Il s'agit d'une patiente âgée de 44 ans qui a consulté au service de dermatologie à la suite d'une éruption cutanée. La patiente a rapporté l'installation depuis un mois de lésions cutanées et depuis 10 jours l'apparition d'une dysphagie ainsi qu'une fatigue de plus en plus intense par ailleurs le patient éprouvait une légère faiblesse musculaire. L'examen clinique trouvait une patient en bon état générale (performans status à 1) et avait objectivé un érythème au niveau du visage et les extrémités (Figure 1, Figure 2) et de la face d'extension des membres, l'examen neurologique était normal, Le bilan biologique a montré des enzymes musculaires élevées notamment les lactates déshydrogénases (LDH). Une biopsie musculaire a été réalisée en faveur d'une myosite. La patiente a était mise sous prednisolone à la dose de 40mg/jour. Une régression des lésions cutanées ainsi que de la fatigue musculaire ont été obtenus sous corticothérapie. Un bilan étiologique ainsi qu'une biopsie du cavum ont permis le diagnostic d'un carcinome indifférencié du cavum. Le bilan d'extension fait d'un scanner thoraco-abdomino-pelvien n'a pas trouvé de métastases à distance permettant ainsi a classe la malade en stade II et la malade a eu une radio chimiothérapie concomittante.

## Discussion

L′association DM et cancer est fréquente allant de 18 à 32% [1], elle peut survenir à n'importe quel âge mais elle touche essentiellement l'adulte. Elle possède deux pics de fréquence: entre 5 et 14 ans pour l'enfant et dans la 5ème ou 6ème décennie chez l'adulte [1]. Tous les types histologiques et toutes les localisations de cancers observés dans la population générale peuvent être associés à la DM Son association avec le carcinome nasopharyngé (NPC) est peu décrite, le plus souvent elle est associe au type undifférencié, un peu moins fréquemment au type peu différencié et jamais au cancer bien différencié [3], elle peut apparaître avant le cancer, en même temps que le cancer ou après le diagnostic de cancer. Son mécanisme pathogénique reste encore mal élucidé. Mais il s'agit d'un dérèglement de l'immunité humorale et cellulaire [3]. Deux théories ont été avancées: hormonale et immunoallergique. Dans la première hypothèse, c'est la tumeur qui secrète des polypeptides hormonaux actifs sur le plan biologique mais inappropriés sur le plan homéostatique. Ces polypeptides seraient responsables des différents syndromes cliniques de type endocrinien. Dans la théorie immuno-allergique, le syndrome paranéoplasique serait le résultat de réaction croisée des anticorps produits contre les antigènes tumoraux, avec les tissus normaux ayant une similitude de structure [3]. Le diagnostic de DM est basé sur cinq critères selon Bohan et Peter [4]: faiblesse musculaire progressive et symétrique des ceintures et des muscles fléchisseurs du cou; des signes dermatologiques (rash héliotrope périorbitaire avec œdèmes, papules Grotton); une biopsie musculaire en faveur de la myosite; une élévation des enzymes musculaires sériques témoignant de la nécrose musculaire Ce constitue un critère diagnostique. La créatine kinase (CPK) représente l'enzyme musculaire la plus spécifique, elle renseigne sur une souffrance objective du tissu musculaire ils sont augmentée dans 75 à 85% des cas, cependant un taux normal ne doit pas écarter le diagnostic; profil électromyographique en faveur d'une atteinte musculaire.

La présence de trois ou quatre de ces critères, en plus de l′éruption cutanée permet de faire le diagnostic de DM, et la présence de deux critères associés à l′éruption cutanée est très suggestive de la DM. La présence de 4 critères a permis de faire le diagnostic dans notre cas. Le type de néoplasie associé aux DM varie selon l’étude et la région géographique du patient. Les cancers gynécologiques notamment le cancer de l'ovaire sont les plus fréquents chez les femmes [5] avec plusieurs cas rapportés dans la littérature sous forme de cas. Chez les hommes, il s'agit plutôt de cancer du poumon, digestifs et de lymphomes [5]. Chan à publié une série de cas de DM suivis à Singapour, dont 41% étaient associés à un cancer, ce dernier relevant dans 60% des cas d'un CNP [6]. Hu et al. Dans une étude statistique de la DM associé au cancer en Chine, trouvaient une incidence de 20,3%, dont 78,4% concernaient le CNP [7]. La DM constitue un facteur pronostique péjoratif du cancer. Le traitement est essentiellement étiologique. Certaines observations ont décrit une amélioration de la dermatomyosite sans immunosuppresseurs après la résection de cancers. Aussi le traitement cytotoxique anti tumoral peut s'accompagner d'une régression de l′inflammation mais il peut induire la progression du cancer. Et l’évolution dépend de celle du néoplasie sous jacente. La DM est corticosensible, Les corticoïdes constituent le traitement de référence et de première ligne des myosites. 60 à 70% des DM répondent à la corticothérapie orale à base de prednisone [8]. L'hydroxychloroquine, comme alternatif aux stéroïdes, se montre assez efficace dans à peu près 80% des patients atteints de DM [9]. La DM associée au carcinome du nasopharynx est rare, dans ce cas l'espérance de vie à 5 et 10 ans selon Hu et al, est de 50% et 34,5% respectivement [7].

## Conclusion

Les syndromes paranéoplasiques, parfois révélateurs de certains UCNT, constituent alors un élément aussi bien de diagnostic que de surveillance des UCNT. Devant une dermatomyosite survenant chez des malades originaires des zones endémiques, le cancer du cavum doit être recherché en priorité.
